# Effect of First Mother's Own Milk Feeding Time on the Risk of Moderate and Severe Bronchopulmonary Dysplasia in Infants With Very Low Birth Weight

**DOI:** 10.3389/fped.2022.887028

**Published:** 2022-05-18

**Authors:** Yiming Zhu, Xiaohui Chen, Jingai Zhu, Chengyao Jiang, Zhangbin Yu, Ailing Su

**Affiliations:** Department of Paediatrics, Women's Hospital of Nanjing Medical University, Nanjing Maternity and Child Health Care Hospital, Nanjing, China

**Keywords:** mother's own milk, breastfeeding, bronchopulmonary dysplasia, infant, very low birth weight

## Abstract

**Objective:**

To explore the effect of mother's own milk (MOM) feeding time on the risk of moderate and severe bronchopulmonary dysplasia (BPD) in infants with very low birth weight (VLBW).

**Methods:**

Clinical data from 630 infants with VLBW were retrospectively analyzed. Participants were divided into the early mother's own milk (EMOM) feeding group (first mother's own milk feeding time ≤72 h after birth, *n* = 397) and the late mother's own milk (LMOM) feeding group (first mother's own milk feeding time >72 h after birth, *n* = 233). Differences in the incidence of moderate and severe BPD among the two groups were analyzed using the chi-square test. Effects of MOM feeding time on the incidence of moderate and severe BPD were evaluated using univariate and multivariate logistic regression analysis.

**Results:**

The incidences of moderate and severe BPD in the EMOM feeding group and the LMOM feeding group were 13.9% (55/397) and 21.0% (49/233), respectively (*P* = 0.019). Variate logistic regression analysis showed that the LMOM feeding group had an increased risk of moderate and severe BPD compared with the EMOM feeding group (OR = 1.656, 95% CI:1.083–2.532). The results of multivariate logistic regression analysis showed that the LMOM feeding group had an increased risk of moderate and severe BPD compared with the EMOM feeding group (OR = 1.894, 95% CI:1.127–3.185).

**Conclusion:**

The first time of MOM feeding within 72 h after birth and the persistence of mother's own milk feeding during hospitalization can reduce the incidence of moderate and severe BPD in infants with VLBW.

## Introduction

Bronchopulmonary dysplasia (BPD) is a common respiratory disease in preterm infants. Due to its many complications, high fatality rate, and lack of effective treatment, it has become an important issue affecting the survival rate and quality of survival of preterm infants, especially in infants with very low birth weight (VLBW). Existing research shows that MOM feeding can reduce the risk of BPD in infants with VLBW (1, 2). In neonatal intensive care units (NICU) in China, human milk sources mainly include two types of MOM and donor milk, among which MOM is the first choice; consequently, it is useful to investigate the factors related to MOM for BPD. Infants with VLBW are usually unable to benefit from their own mother's milk. According to a study on influencing factors of MOM in China in 2019, the survival rate in low weight exposure after birth (OR.57, 95% CI:0.39–0.84) was significantly lower than in non-low weight infants (3). In the study of 601 infants with VLBW, the proportion of MOM in the first week was the lowest of all stages during hospitalization. When infants with VLBW receive MOM in NICU depends on the infant's condition, but it is also mainly related to the maternal postpartum lactation condition (4). If infants with VLBW are able to feed naturally and maternal lactation initiation time was normal, infants with VLBW can theoretically receive the first MOM within 72 h of birth.

## Materials and Methods

### Participants

This is a retrospective analysis of the infants with VLBW admitted to the Department of Pediatrics, Women's Hospital of Nanjing Medical University/ Nanjing Maternity and Child Health Care Hospital from January 2015 to December 2019.

Inclusion criteria: (1) birth weight <1,500 g and gestational age at birth <34 weeks; (2) admission within 24 h after birth; and (3) intestinal nutrition within 72 h after birth, and mother's own milk feeding accounted for >90% of participants.

Exclusion criteria: (1) hospital day ≤ 14 days; (2) non-cured infants who did not reach 28 days, lost after discharge; (3) congenital malformation or genetic metabolic diseases; and (4) incomplete data.

### Methods

The aim of this study was to provide a retrospective analysis of the effect of MOM feeding time on the risk of moderate and severe bronchopulmonary dysplasia (BPD) in infants with VLBW.

#### Data Collection

Paper or electronic scanned versions of the medical records of infants with VLBW admitted to the NICU of Nanjing Maternity and Child Health Hospital, affiliated to Nanjing Medical University, from January 2015 to December 2019 were used. The NingBX neonatal homogeneity platform (http://www.ningbx.com) was an online database where we got the infants' information from. The hospital established a human milk bank in August 2013. All nutrition types and feeding volumes of infants included in the study were recorded in the nursing list. The variables in this study included sex, gestational age, birth weight, delivery, polycyesis, 1-min Apgar score, antenatal steroid, surfactant use, mechanical ventilation (MV), time on MV ≥7days, and total MOM feeding. Neonatal outcomes examined included the incidences of BPD, necrotizing enterocolitis (NEC), retinopathy of prematurity (ROP), grade III or IV intraventricular hemorrhage (IVH III/IV), sepsis, late-onset sepsis (LOS), neonatal respiratory distress syndrome (NRDS), and patent ductus arteriosus (PDA).

#### Grouping

Infants with VLBW were divided into the EMOM feeding group and the LMOM feeding group, according to the classification criteria.

### Definitions

Bronchopulmonary dysplasia was defined as the need for supplementary oxygen for ≥28 days and classified as mild, moderate, or severe BPD following the 2005 consensus (5). NEC and severity grades of NEC were defined according to Bell's stage, more than stageII (6). International Classification of ROP (ICROP) was defined as ≥stage 2. IVH was defined as grade III or IV intraventricular hemorrhage. LOS was diagnosed by the presence of clinical signs of sepsis and confirmed by blood culture after 3 days of life. PDA was defined by echocardiographic examination, systolic or continuous murmur, bounding pulse or hyperactive precordial pulsation, hypotension, and respiratory difficulty. EMOM feeding was defined as feeding time <72 h after birth. LMOM feeding was defined as feeding time >72 h after birth. First enteral feeding was defined as more than 90% of the MOM, and the rest can be formula milk or donor milk.

### Statistical Analysis

Statistical analysis was performed using SPSS 26.0 software (Armonk, NY: IBMCorp). The median and interquartile range represent skewed variables; and frequencies and percentages represent categorical variables. The chi-square test and the Kruskal–Wallis test were used to compare the risk of BPD with neonatal data and clinical information. Univariate logistic regression was used to analyze the relationship between related variables and moderate and severe BPD occurrence; multivariate logistic regression analyzed the relationship between first mother's own milk feeding time and BPD occurrence, adjusting for risk factors closely associated with outcome. The risk was reported as an odds ratio (OR) with 95% CI. All tests were two-sided tests with *P* ≤ 0.05 deemed significant.

## Results

### General Information and Treatment Conditions

**Figure 1:**
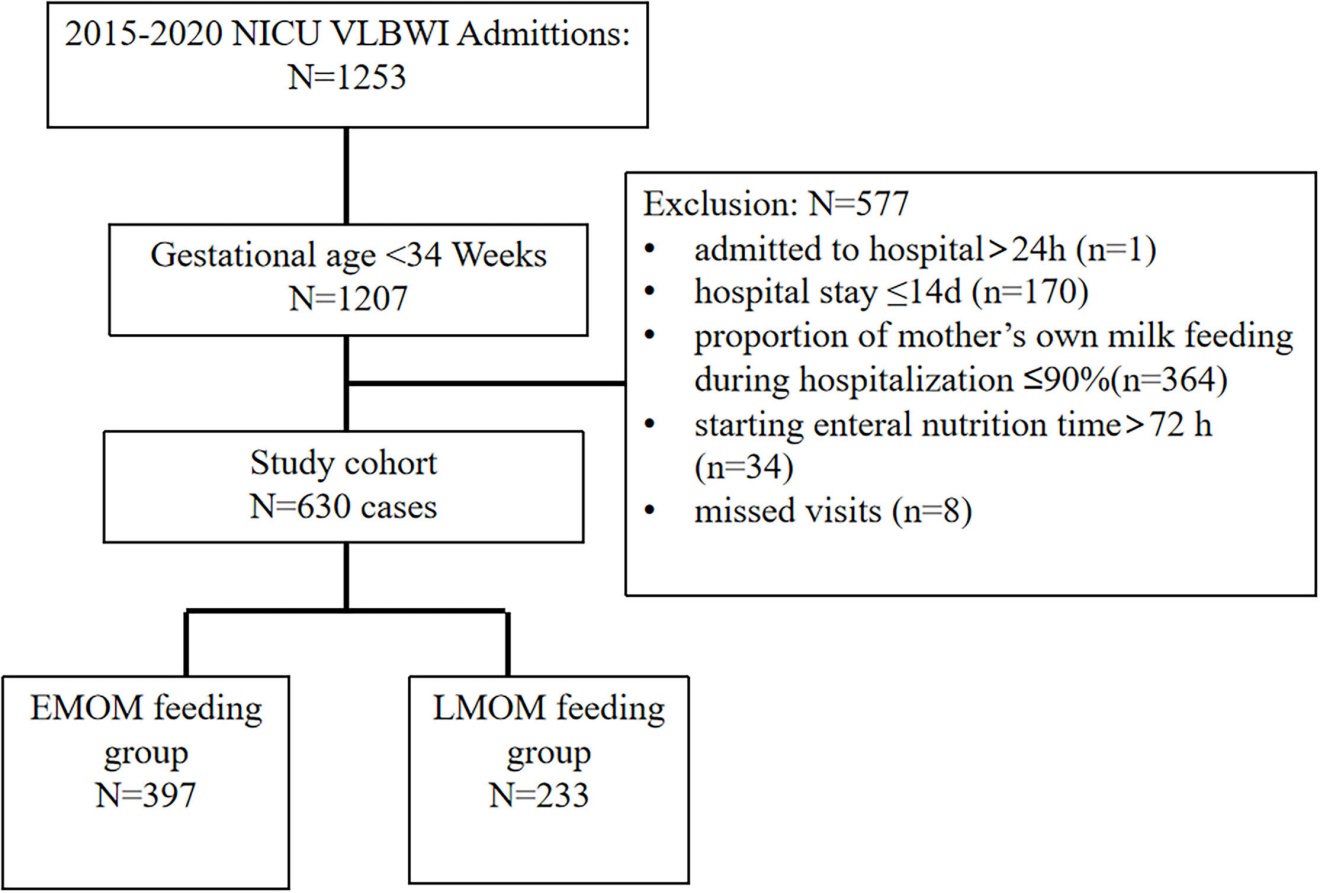
Flowchart of infants included in this study. VLBWI, very low birth weight infants.

A total of 1,253 infants with VLBW were recruited during this study. Only 630 infants met the criteria; the inclusion process was detailed in Figure 1. There was a significant difference between the two groups in total MOM feeding during hospitalization (*P* < 0.05). There were no significant differences between gestational age, birth weight, cesarean section, 1-min Apgar score <7, prenatal hormone use, surfactant use, MV, and time on MV ≥7 days, (*P* > 0.05) (Table 1).

**Table 1:** Clinical characteristics and treatment (Example [%]).

	**EMOM feeding group *N* = 397**	**LMOM feeding group *N* = 233**	***Z/*** **χ2 value**	***P*** **value**
**Peripheral factors**				
Cesarean section	213 (53.7)	128 (54.9)	0.097	0.755
Multiple gestation	77 (19.4)	63 (27.0)	4.963	0.026
Antenatal steroid	365 (91.9)	204 (87.6)	3.230	0.072
**Neonatal factors**				
Male	192 (48.4)	134 (57.5)	4.921	0.027
Gestational age, weeks [*M*(*Q*_1_, *Q*_3_*)*]	29.1 (28.1, 30.3)	29.3 (28.3, 30.7)	−1.678	0.093
Birth weight, g [*M*(*Q*_1_, *Q*_3_*)*]	1210 (1085, 1350)	1200 (1070, 1350)	−0.021	0.983
1-min Apgar <7	52 (13.1)	31 (13.3)	0.005	0.941
**Treatment**				
Surfactant use	273 (68.8)	164 (70.4)	0.181	0.670
MV	109 (27.5)	66 (28.3)	0.055	0.814
MV ≥7days	40 (10.1)	31 (13.3)	1.531	0.216
Average time of start feeding[*M*(*Q*_1_, *Q*_3_*)*]	25.9 (22.1, 30.8)	26.2 (22.3, 30.9)	−0.633	0.527
Total MOM feeding [mL, *M*(*Q*_1_, *Q*_3_*)*]	6741.0 (4814.5, 9012.0)	5986.0 (4148.0, 8500.5)	−2.364	0.018

*Total mother's own milk feeding refers to the total mother's own milk feeding amount during hospitalization*.

### Complications

The incidence of moderate and severe BPD was lower in EMOM feeding (*P* < 0.05), and no significant differences were found in NEC (≥ stage 2), ROP, IVH III/IV, sepsis, LOS, NRDS, and PDA (*p* > 0.05) (Table 2).

**Table 2:** Comparisons of the occurrence of VLBW infants between the two groups (Example [%]).

**Group**	***N***	**Moderate** **and severe BPD**	**NEC (≥stage 2)**	**ROP**	**IVH III/IV**	**sepsis**	**LOS**	**NRDS**	**PDA**
EMOM feeding group	397	55 (13.9)	18 (4.5)	95/307 (30.9)	40 (10.1)	153 (38.5)	51 (12.8)	366 (92.2)	283 (71.3)
LMOM feeding group	233	49 (21.0)	11 (4.7)	58/177 (32.8)	27 (11.6)	87 (37.3)	27 (11.6)	216 (92.7)	154 (66.1)
χ2 value		5.486	0.012	0.173	0.353	0.09	0.214	0.055	1.861
*P* value		0.019	0.914	0.678	0.552	0.765	0.643	0.815	0.172

## Univariate and Multivariate Logistic Regression Analysis of First Mom Feeding Time and Risk of Moderate and Severe Bpd Occurrence

### Uni-Variate Analysis

There was a higher risk in the LMOM feeding group, with an OR value of 1.656 (95%CI: 1.083–2.532) (Table 3).

**Table 3:** Univariate logistic regression analysis of the factors affecting moderate and severe BPD.

	***SE*** **value**	***Wald*** **value**	***P*** **value**	***OR*** **value (95%CI)**
Male	0.217	1.757	0.185	1.333 (0.871–2.039)
Gestational age increased	0.073	54.706	0.000	0.582 (0.504–0.672)
Birth weight increased	0.001	66.837	0.000	0.995 (0.994–0.996)
Cesarean section	0.220	11.968	0.001	0.467 (0.304–0.719)
Multiple gestation	0.240	4.093	0.043	1.625 (1.015–2.600)
Surfactant use	0.310	17.335	0.000	3.642 (1.982–6.691)
MV	0.234	76.994	0.000	7.811 (4.935–12.363)
MV ≥ 7days	0.297	109.275	0.000	22.266 (12.444–39.840)
Total MOM feeding	0.000	91.762	0.000	1.000 (1.000–1.000)
sepsis	0.224	31.548	0.000	3.514 (2.266–5.447)
LOS	0.293	2.745	0.098	1.626 (0.915–2.888)
NRDS	0.730	4.718	0.030	4.887 (1.168–20.459)
PDA	0.332	20.390	0.000	4.473 (2.335–8.570)
LMOM feeding	0.217	5.418	0.020	1.656 (1.083–2.532)

### Multi-Variate Analysis

Screening factors were statistically significant as independent variables together with the LMOM feeding group, which showed that the morbidity rate in moderate and severe BPD was higher than that in the EMOM feeding group with an OR value of 1.894 (95% CI: 1.127–3.185) (Table 4).

**Table 4:** Multivariate logistic regression analysis of factors affecting moderate and severe BPD.

	**β value**	***SE*** **value**	***Wald*** **value**	***P*** **value**	***OR*** **value (95%CI)**
Gestational age increased	−0.210	0.100	4.421	0.035	0.811 (0.666–0.986)
MV ≥7 days	2.372	0.336	49.686	0.000	10.718 (5.542–20.727)
sepsis	0.579	0.273	4.484	0.034	1.784 (1.044–3.047)
PDA	0.859	0.356	5.829	0.016	2.360 (1.175–4.739)
LMOM feeding	0.639	0.265	5.810	0.016	1.894 (1.127–3.185)

*Cox and Snell R^2^ = 0.217, Nagelkerke R^2^ = 0.366*.

## Discussion

Bronchopulmonary dysplasia is a common respiratory complication affecting the survival rate and quality of survival of infants with VLBW. Because infants with moderate and severe BPD still need to use oxygen at specific times, it often affects the severity of the disease and prognosis, which not only is the focus of clinicians but also affects the treatment plan.

An observational cohort study of 16,407 infants with VLBW in five countries showed that the incidence of moderate and severe BPD in infants with VLBW was 15.7% (2,580/16,407) (7). A multi-center retrospective study collecting annual clinical data from 19 units in 2017 showed that the incidence of VLBW infants that suffer from moderate and severe BPD was 6.6% (51/768) (8). Five-year data collected from this study showed that the incidence of moderate and severe BPD in infants with VLBW was 16.5% (104/630), which is higher than that reported in other studies, both at home and abroad, and may be related to single centers, study duration, and inclusion and exclusion criteria defined in the study. The results of the multivariate logistic regression analysis in this study showed that 7 days of mechanical ventilation, sepsis, and PDA were all independent risk factors for moderate and severe BPD, which is consistent with other relevant studies (9–11). The incidence of BPD decreased with increasing gestational age (12), and the results of this study show that gestational age growth also helps reduce the occurrence of moderate and severe BPD.

Research primarily focused on the occurrence of BPD; however, the relationship between first MOM feeding time and the occurrence of moderate and severe BPD has not been reported. This study found that first MOM feeding time in infants with VLBW >72 h after birth was a high-risk factor for moderate and severe BPD, suggesting that MOM feeding within 72 h of the birth of infants with VLBW reduces the risk of moderate and severe BPD. Breast milk reduces oxidative stress and provides antioxidant protection, vitamin A, and scavenger enzymes, which play an important role in reducing oxidative damage, protecting, and promoting immune system maturation in premature infants (13, 14). However, oxidative stress and inflammatory immune response are related to the occurrence and development of BPD (15, 16). Equally, infection is one of the risk factors for moderate and severe BPD (9), and breastfeeding has been confirmed to reduce the occurrence of many common complications in premature infants, such as sepsis, which not only is conducive to improving the systemic condition of premature infants but also reduces the risk of hospital infection and thus partly reduces the rate of moderate and severe BPD (17).

The preventative role of breast milk for BPD has been confirmed by systematic review and meta-analysis (18), but no randomized controlled trials have verified whether maternal or donor milk is best for BPD prevention. An observational multi-center study from France showed feeding very preterm infants with their mother's own fresh milk was associated with a reduced risk of BPD (19). Some studies have found that maternal breastfeeding accounts for every 10% increase and that the risk of BPD decreases by 9.5% (20), so maternal breastfeeding may be one of the potential confounding factors in this study. Due to uncontrollable external factors, as well as the child's condition, it cannot be guaranteed that the feeding type is MOM feeding. Therefore, this study limited the inclusion of MOM feeding hospitalization ratio >90% to control the proportion of MOM feeding and indirectly eliminate, if possible, their bias on the outcome.

The particularity of maternal lactation with premature infants affects the time when some premature infants receive maternal breast milk. Preterm birth is an independent risk factor for delayed lactation initiation (21). The results of a study in China show that the incidence of premature lactation initiation delay in premature infants is 36%, which is significantly higher than that of full-term infants (22). The reasons mainly include an immature lactation system, the lack of infant sucking stimulation, and psychological factors, among others (23). Since lactation initiation is the initial period of mass secretion of breast milk if the puerperant has normal lactation initiation, infants with VLBW can theoretically breastfeed within 72 h. In this study, all the included infants with VLBW were eligible for enteral nutrition within 72 h, and different timings of first maternal breastfeeding were excluded. Study data showed that infants with VLBW of the LMOM feeding group received maternal breastfeeding later than 72 h after birth, mainly due to no lactation or insufficient lactation due to delayed maternal lactation, no normal lactation to NICU in time, and medical staff failing to feed after breastfeeding. For maternal separation of mother and infant, electric breast suction in the early postpartum stage helps to advance lactation initiation and lactation maintenance (24, 25). China's recommendations issued in 2016 in NICU state that premature infants in NICU prefer maternal breastfeeding and women should be encouraged to start breastfeeding half an hour after delivery. Fresh colostrum should be delivered immediately to NICU to feed preterm infants (26). The pediatrician should follow the guidelines and carry out education, emphasize the importance of maternal milk to the mother and her family, and enhance their enthusiasm for milk delivery. The department should set up relevant feeding standards based on the guidelines and provide maternal breast milk to hospitalized infants.

The results provide a practical basis for promoting active breast sucking and instant milk feeding in infants with VLBW, while highlighting the importance of the time for first MOM feeding of infants with VLBW. MOM feeding of infants with VLBW within 72 h can reduce the occurrence of moderate and severe BPD. Early exposure to various beneficial components in maternal breast milk can promote the maturation of the immune system and improve the body's antioxidant capacity. However, when the time was further reduced, the difference was not statistically significant between the two groups, which may relate to insufficient sample size and uneven distribution of age at the time of the first maternal breastfeeding. The results showed a higher maternal milk intake during hospitalization than the LMOM feeding group, although the occurrence of moderate and severe BPD was independent of maternal breast milk aggregates during hospitalization. Earlier MOM seems to indicate that infants with VLBW can obtain more adequate maternal milk supply during hospitalization, partly because normal lactation starts to reduce the risk of insufficient lactation and partly because early delivery shows that the mother can actively pump milk and chooses to participate in the breastfeeding of the infant with VLBW; these factors complement each other and jointly improve MOM feeding time of infants with VLBW.

## Limitations

There are some limitations to this study. First, it is a single-center study with limited data that needs confirmation through multi-center studies; second, there are many factors affecting BPD, and we only selected some related factors, despite the limited number, it can still illustrate the protective effect of early mother's own milk feeding on BPD, which can also be easily understood clinically; finally, the 72-h criterion was subjective, and more data is needed to verify this time period.

## Conclusions

In conclusion, the first MOM feeding time within 72 h after birth and the persistence of MOM feeding during hospitalization can reduce the incidence of moderate and severe BPD in infants with VLBW. Hospitals and medical staff should follow guidelines, which can improve the enthusiasm of maternal milk absorption by maternal separation, and give infants with VLBW MOM feeding in good time. More multi-center large-sample clinical trials are needed to validate the outcomes of the relationship between first MOM feeding time and infants with VLBW.

## Data Availability Statement

The raw data supporting the conclusions of this article will be made available by the authors, without undue reservation.

## Ethics Statement

The studies involving human participants were reviewed and approved by the Ethics Committee of the Maternity Hospital Affiliated to Nanjing Medical University. Written informed consent to participate in this study was provided by the participants' legal guardian or next of kin.

## Author Contributions

YZ and AS conceptualized and designed the study, drafted the initial manuscript, reviewed, revised the manuscript, supervised data collection, and critically reviewed the manuscript for important intellectual content. XC, JZ, and CJ collected data and carried out the initial analyses. All authors approved the final manuscript as submitted and agreed to be accountable for all aspects of the work.

## Funding

This work was supported by the Six Top-Notch Talent-funded Projects (LGY 2019008), the Nanjing Medical Science and Technology Development Fund (ZKX 19045), and the Nanjing Medical University's Specialized Disease Cohort -funded Project (NMUC 2020037).

## Conflict of Interest

The authors declare that the research was conducted in the absence of any commercial or financial relationships that could be construed as a potential conflict of interest.

## Publisher's Note

All claims expressed in this article are solely those of the authors and do not necessarily represent those of their affiliated organizations, or those of the publisher, the editors and the reviewers. Any product that may be evaluated in this article, or claim that may be made by its manufacturer, is not guaranteed or endorsed by the publisher.
